# Evolution of population genetic structure of the British roe deer by natural and anthropogenic processes (*Capreolus capreolus*)

**DOI:** 10.1002/ece3.430

**Published:** 2013-01-10

**Authors:** Karis H Baker, A Rus Hoelzel

**Affiliations:** School of Biological and Biomedical Sciences, Durham UniversitySouth Road, Durham, DH1 3LE, UK

**Keywords:** Conservation genetics, deer, population bottleneck, population structure, translocation

## Abstract

Human influence typically impacts on natural populations of conservation interest. These interactions are varied and sometimes complex, and may be negative and unintended or associated with conservation and management strategy. Understanding the details of how these interactions influence and are influenced by natural evolutionary processes is essential to the development of effective conservation strategies. In this study, we investigate a species in Britain that has experienced both negative impact through overhunting in historical times and management efforts through culls and translocations. At the same time, there are regional populations that have been less affected by human influence. We use mtDNA and nuclear microsatellite DNA markers to investigate patterns of connectivity and diversity and find multiple insular populations in Britain that probably evolved within the Holocene (when the habitat was free of ice). We identify three concurrent processes. First, surviving indigenous populations show highly provincial patterns of philopatry, maintaining and generating population structure on a small geographic scale. Second, founder populations into habitat extirpated of native populations have expanded, but remained largely insular. Third, introductions into established populations generate some admixture. We discuss the implications for the evolution of diversity of the integration of natural processes with anthropogenic influences on population size and distribution.

## Introduction

Natural processes associated with vicariance, habitat dependence, local adaptation, and dispersal strategies (evolved to maximize fitness) promote the distribution of diversity among conspecific populations. These processes lead to the evolution of population structure over varying geographic scales and eventual speciation. At the same time, anthropogenic activities are often superimposed, and the impact on population structure will depend on how these different processes interact. Humans can cause fragmentation, population declines, expansions (e.g., when non-native species are introduced), and extirpation.

For populations that have been influenced by declines, recovery in population size can be rapid, but there may be a long-term impact on diversity (both through the initial loss of allelic diversity and through drift over time). Typically, population size recovery can occur as a species naturally disperses and recolonizes formerly occupied areas. This process of natural recolonization usually occurs when degraded habitats are restored and dispersal corridors are available (see Hochkirch et al. 2007). Populations may also be restored through human intervention using translocations. There are various complications associated with this process including the loss of diversity and distortion of allele frequencies if the founder population size used for the translocation is small (e.g., Ralls et al. 2000), and the possibility that interbreeding between introduced and native populations may result in reduced fitness if too dissimilar (e.g., Rhymer and Simberloff 1996). At the same time, translocations are often proposed as a means of “genetic rescue” introducing new genes into depauperate threatened or endangered populations (e.g., Pimm et al. 2006; cf. Creel 2006).

Introduced populations may remain relatively isolated with low diversity following small reintroductions. This was found to be the case for the Alpine ibex, *Capra ibex ibex* where founder populations had mostly been serially bottlenecked (Biebach and Keller 2009), and the white-tailed deer, *Odocoileus virginianus,* where multiple source populations introduced into an area where the species had been extirpated retained founder signatures and genetic structure (DeYoung et al. 2003). However, introduced populations that rapidly expand may retain more variation and integrate with native populations more readily (e.g., Zenger et al. 2003). This should also depend on the interaction between population density, dispersal behavior and range, and proximity to native populations (see Latch and Rhodes 2005). In this study, we investigate the effects of historical population declines followed by more recent recolonization via natural and non-natural dispersal among populations of the British roe deer (*Capreolus capreolus*). This set of population histories provides an opportunity to investigate the implications of the interaction between natural processes associated with population expansion and philopatry, and the influence of anthropogenic impacts on population size and distributions.

The only deer species indigenous to the United Kingdom are the roe deer and red deer (*Cervus elaphus*). The first postglacial records of roe deer date back to between 10,050 and 9600 YBP from a site found at Thatcham in Berkshire (Yalden 1999). During the late medieval period, British roe deer populations were severely reduced, probably as a result of overhunting and deforestation. Historical documents indicate that these declines were so severe that roe deer were confined to parts of Scotland and possibly some of the northern English border counties (Whitehead 1964). In most of the midlands and southern English counties, roe deer were reportedly absent by the 16th century (Ritson 1933). During the 1800s, roe deer populations began to recover and, since then, this recovery has been remarkable (see Ward 2005). Recovery in northern parts of the United Kingdom can generally be attributed to natural expansion of remnant populations into formerly occupied areas following afforestation (Taylor 1948). In southern parts of the United Kingdom, all populations are believed to have descended from reintroduction events (see Table 1 and Whitehead 1964). All populations in the United Kingdom have now expanded substantially in size, and numbers are currently controlled in culls managed by either independent landowners or local collaboratively run Deer Management Groups (Phillips et al. 2010). The series of independent (from European founders) and compounded bottlenecks from translocated populations within the United Kingdom, together with the overall impact of the medieval bottleneck, could be expected to have reduced genetic diversity. Depending on factors associated with dispersal behavior and population dynamics, as indicated above, more or less isolation and structure may have evolved. One previous study examined roe deer population genetics in the United Kingdom using allozyme markers, and showed that roe deer exhibited polymorphism at only one locus, consistent with the expectation of reduced diversity. The one polymorphic locus indicated evidence of an east/west cline in southern populations, which was described as consistent with the reintroduction records of roe deer (Hewison 1995). However, the resolution of that study was low, given the small number of markers applied, and their relatively low diversity levels.

**Table 1:** Summary of all known successful roe deer introductions into mainland Britain (after Whitehead 1964)

Site of introduction	Date	Site of origin	Number released
Southern introductions			
Milton Abbas, Dorset	1800	Perth, Scotland	4
Abbotsbury, Dorset	1820	Unknown	
Windsor Great Park, Berks	1825	Dorset	4
Windsor Great Park, Berks	1850	Petworth	
Epping Forest, Essex	1883	Dorset	6
Epping Forest, Essex	1884	Unknown	8
Thetford, Norfolk	1884	Württemberg, Germany	12
Petworth, Sussex	1800	Unknown	
Petworth, Sussex	1890	Scotland	
Brentwood, Essex	1892	Unknown	2
Horsham, Sussex	1931	Unknown	
Northern introductions			
Maybole, Strathyclyde, Scotland	1820	Unknown	
Annandale, Dumfries, Scotland	1854	Unknown	
Drumlanrig, Dumfries, Scotland	1860	Unknown	
Windermere, Cumbria	1913	Austria	12

In this study, we investigate roe deer populations sampled from areas in the northern and southern United Kigndom (see Table 2) using 16 polymorphic microsatellite DNA markers together with 744 bp sequence data from the mtDNA control region. These areas were chosen as to best represent the differing population histories (as described above). We test the hypotheses that admixture among native and introduced populations will have resulted in recovered diversity following the historical bottleneck, and minimized population structure among rapidly expanding populations over the restricted geographic range represented by mainland Britain. These are realistic expectations, given the known histories and rate of expansion. The very different reality of high levels of structure and insularity may depend on strong philopatric behavior and the way in which natural populations are founded. We consider the results in the context of how the contemporary processes of philopatry and dispersal have interacted with historical processes to generate the current pattern of differentiation.

**Table 2:** Regions, counties, and locations with number (*n*) of roe deer samples collected from across the United Kingdom used for microsatellite, mitochondrial DNA

Region	Area	Mitochondrial samples (*n*)	Microsatellite samples (*n*)
Scotland	Moray	29	39
	Perth	34	39
	Glasgow	–	9
	Ayrshire	51	59
North West	Carlisle	28	29
	Lancashire	13	18
North East	Durham	11	17
	North Yorkshire	25	29
South East	Norfolk	40	44
South West	Berks	20	18
	Dorset	39	39
	Wiltshire	7	7
	Somerset	17	20
Total		314	367

## Methods

### Sampling and DNA isolation

**Figure 1:**
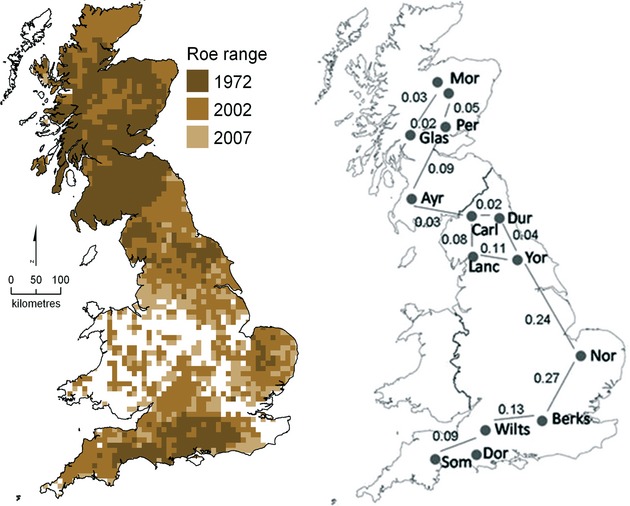
Census data mapping presence in 10 km square regions for roe deer across Britain for 1972, 2002, and 2007. Panel to right shows *F*_ST_ comparisons from microsatellite DNA data. Census map figure reprinted with permission from British Deer Society report by A. I. Ward.

Tissue samples were taken from 367 culled roe deer (126 males, 230 females, and 11 unknown sex) from 14 main sampling areas across the United Kingdom during 2007–2009 (see Table 2; Fig. 1) and stored in 20% DMSO/saturated NaCl solution (Amos and Hoelzel 1991). For a number of analyses, sample sets were divided according to their location in the north or the south. Northern locations were defined as those in Scotland and northern England (i.e., sites to the north from Yorkshire and Lancashire, north of 52°N latitude; see Fig. 1) and southern sites as those south from Norfolk; see Figure 1. Total genomic DNA was extracted from samples using a proteinase K digestion procedure followed by the standard phenol–chloroform method and stored at −20°C.

### Amplification and genotyping of microsatellites

Individuals were genotyped via polymerase chain reaction (PCR) at 18 previously published microsatellites shown to be polymorphic in roe deer: BM1706, BM757, BM848, CSSM39, CSSM41, CSSM43, HUJ1177, IDGVA29, IDVGA8, NVHRT48, BMC1009, OarFCB304 (Galan et al. 2003), ILST011, MAF70 (Crawford et al. 1995), MCM505, MCM131 (Hulme et al. 1995), NVHRT24 (Roed 1998), and RT1 (Wilson et al. 1997). Microsatellites loci were multiplex amplified using Qiagen™ Multiplex kit. (Qiagen, West Sussex, UK) Primer sequences, details of the multiplex mixes and PCR reaction conditions are shown in Table S1. PCR products were genotyped on a 3730 ABI DNA Analyser (DBS Genomics, Durham, UK) and visualized with Peak scanner™ software v 1.0 (Applied Biosystems, Foster City, California). Microsatellite loci were tested for null alleles, large allele dropout, and scoring errors due to stutter peaks using MICROCHECKER 2.2.3 (Van Oosterhout et al. 2004). Deviations from Hardy–Weinberg equilibrium (HWE) were tested for each population and each locus using the Markov chain method proposed by Guo and Thompson (1992), implemented in the software ARLEQUIN 2.000 (Schneider et al. 2000). Tests for linkage disequilibrium were carried out for each pair of loci using an exact test based on a Markov chain method as implemented in Genepop 3.4 (Raymond and Rousset 1995).

### Amplification and sequencing of mitochondrial DNA

A mtDNA control region fragment of 744bp was amplified using the two primers developed by Randi et al.(1998): Lcap Pro 5′-CGT CAG TCT CAC CAT CAA CCC CCA AAG-3′ and Hcap Phe 5′-GGG AGA CTC ATC TAG GCA TTT TCA GTG-3′. PCR reactions (20 μL) contained 0.2 pmol/L/μL each primer, 0.2 mmol/L each dNTP, 10 mmol/L Tris-HCL, pH 9.0, 1.5 mmol/L MgCl_2_, and 0.4 units of *Taq* polymerase (New England Biolabs, Hitchin, UK) with cycle conditions: 95°C for 5 min; 35 cycles at 94°C for 45 sec, 51°C for 45 sec; and 72°C for 45 sec; 72°C for 5 min. PCR products were purified using Qiagen columns (Qiagen, Inc) and directly sequenced using an ABI 377 automated sequencer. All sequences were aligned using Clustal X (Larkin et al. 2007).

### Genetic diversity and structure

For mtDNA data, the program DNA sp 10.4.9 (Rozas et al. 2003) was used to calculate mitochondrial DNA polymorphism estimated as haplotypic diversity (*H*; Nei and Tajima 1981), nucleotide diversity (π, Nei 1987), and average pairwise nucleotide divergence (*k*). For microsatellite DNA data, allelic richness for each locus and population and *F*_IS_ were calculated using the program FSTAT 2.9.3 (Goudet 2001). The sequential Bonferroni method was used to correct for type 1 errors (Rice 1989). The relationship among haplotypes was examined by constructing median joining Networks (Bandelt et al. 1999) implemented in the program NETWORK 3.1.1.1. To determine the level of genetic differentiation between pairs of populations, *F-statistics* (Weir and Cockerham 1984) were calculated for mtDNA and microsatellite DNA loci using ARLEQUIN v 2.0 (Schneider et al. 2000). Two different *F-statistics* were used: a measure that incorporates mtDNA sequence divergence (Φ_ST_) and a measure based on mtDNA haplotype frequencies or microsatellite allele frequencies (*F*_ST_). Significance was tested using 1000 permutations and Bonferroni corrected for multiple comparisons. Distributions of mtDNA haplotypes were examined within subpopulations by plotting haplotypes (excluding singletons) onto a location map of the United Kingdom where samples were collected.

In order to see whether differences in haplotype distributions across the United Kingdom could define population structure, a spatial analysis of molecular variance in the SAMOVA software (Dupanloup et al. 2002) was applied. SAMOVA was run successively with a different *K* (the putative number of populations), ranging from 2 to 10. Analyses were run twice for each value of *K* to check consistency between runs. For each run, 100 simulated annealing processes were performed. The composition of the *K* groups was identified by observing the maximum *F*_CT_ index (the proportion of total genetic variance due to differences between groups of populations; Dupanloup et al. 2002).

The program STRUCTURE 2.0 was used to assign putative number of populations (*K*) based on microsatellite DNA data (Pritchard et al. 2000). Two approaches were used to choose *K*. First, Δ*K*, a measure of the second order rate of change in the likelihood of *K* (Evanno et al. 2005), was calculated to assess the highest hierarchical level of structure. Second, posterior probabilities for the values of *K* with the highest Ln *P*(*X*^|^*K*) were compared. Five independent runs for each *K* value (2–11) were performed at 10^6^ Markov chain Monte Carlo (MCMC) repetitions and 10^5^ burn-in using no prior information and assuming correlated allele frequencies and admixture. The posterior probability was then calculated for each value of *K* using the estimated log likelihood of *K* to choose the optimal *K*. Apparent structure not detected at the higher hierarchical level was reassessed by rerunning the program with subsets of samples.

STRUCTURE was also used to identify migrants and those individuals with migrant or mixed ancestry. Prior population information can be incorporated in an attempt to determine migrant individuals, allowing the program to calculate posterior probabilities that individuals belong to their sampled locality. Structure was run using the usepopinfo option with *K* = 7 and a range of migration rate (MIGPRIOR) values (0.001–0.1). Burn-in and run lengths were the same as for runs without prior population information. Spatially explicit information on population structure was assessed using the program Geneland (Guillot et al. 2005). Although STRUCTURE also has a spatially explicit function, the application in Geneland typically has more power and was therefore included to assess fine-scale patterns of structure. Runs were performed for 100,000 MCMC repeats and replicated 8 times assuming correlated allele frequencies. Populations in the north and south were assessed independently. Postprocessing analysis included an assessment of admixture (based on 100,000 repeats and a burn-in of 200).

Patterns of microsatellite differentiation were visualized using a factorial correspondence analysis (FCA) implemented in GENETIX 4.0 (Belkhir et al. 2000), which gives a visual representation of individual genotype clustering (She et al. 1987). Relationships between geographic distance and genetic distance (based on microsatellite DNA loci) were assessed with a Mantel test (10,000 permutations) using Genepop (Raymond and Rousset 1995).

## Results

### Genotypes

Two microsatellites (IDVGA-29 and MCM131) were excluded from all analyses because of genotyping error revealed by Microchecker. No evidence of genotyping errors was found for any further loci. The test of genotypic disequilibrium for each pair of the 16 microsatellite loci over all populations gave 11 significant values (*P* ≤ 0.05) for 224 comparisons (14 significant values are expected by chance at the 5% level). After Bonferroni correction, six combinations were significant (*P* < 0.0031) at the experimental level; three of these occurred in the Norfolk population. Despite these differences, no clear patterns across samples were observed.

### Genetic diversity

**Figure 2:**
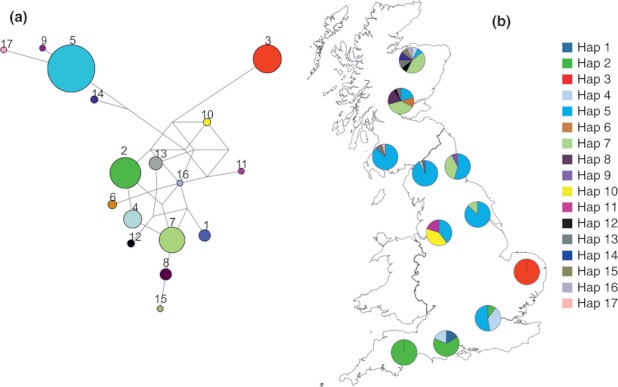
(a) Median joining network of phylogenetic relationships among modern mitochondrial haplotypes where the size of the circle indicates relative frequency of the haplotype. Haplotypes represented are based on 744 base pairs of the mt-DNA d-loop and exclude singletons. (b) Modern roe haplotypes (excluding singletons) and their distributions across the United Kingdom.

Mitochondrial DNA analyses revealed a total of 27 haplotypes and 22 variable sites (18 transitions and 4 transversions). The haplotypes occurred between one and 115 times (distributions are represented in Table S2). Haplotypic diversity (Table 3, Fig. 2) was greatest in the northern sampling sites, especially in Scotland, and lowest at the introduced population in Norfolk, where only one haplotype was found among 40 samples. The mean across all populations was 0.81. Microsatellites were highly polymorphic showing an average of 10.06 alleles per locus, and allelic richness was relatively constant ranging from 3.21 in Somerset to 5.03 in Glasgow (Table 3). Genetic variation expressed as mean *H*_e_ was 0.65 (range 0.59–0.76) and mean *H*_o_ was 0.62 (range 0.49–0.74) with mostly small, positive *F*_IS_ values (Table 3). Genetic variability based on microsatellite DNA loci diminished from north to south within the United Kingdom (range in Scotland: *H*_e_ = 0.72–0.76; middle England: 0.65–0.68; southern England: 0.57–0.64).

**Table 3:** Mitochondrial (mtDNA) control region and microsatellite diversity statistics for roe deer samples at each location

	mtDNA				Microsatellites					
		
	Hap	*H*	π	*k*	A	AR	*F*_IS_	*H*_*o*_	*H*_*e*_	*P*
Moray	9	0.81	0.0044	3.19	7.00	4.57	0.091	0.64	0.70	0.005
Perth	7	0.78	0.0044	3.3	6.25	4.68	0.034	0.69	0.72	0.01
Glasgow	–				5.25	5.03	0.003	0.74	0.76	0.523
Ayrshire	7	0.36	0.0024	1.78	5.81	4.05	0.028	0.64	0.66	0.004
Cumbria	3	0.21	0.0008	3.1	4.75	3.77	−0.044	0.67	0.65	0.92
Lancashire	4	0.82	0.0041	0.56	5.00	4.39	0.053	0.63	0.68	0.144
Durham	3	0.6	0.0042	1.84	4.44	3.77	0.064	0.59	0.65	0.109
N York	5	0.29	0.0025	3.6	3.94	3.33	0.013	0.58	0.60	0.252
Norfolk	1	0.00	–	–	4.19	3.34	0.145	0.49	0.59	0
Berks	3	0.61	0.0038	2.83	4.19	3.61	−0.064	0.65	0.62	0.953
Dorset/Wilts	3	0.53	0.0018	1.3	4.69	3.58	0.009	0.59	0.60	0.788
Somerset	1	0.00	–	–	3.75	3.21	0.047	0.52	0.57	0.0013
Average	4.2	0.81	0.0057	4.23	4.94	3.94	0.0316	0.62	0.65	0

Hap, number of haplotypes; *H*, haplotypic diversity; π, nucleotide diversity; *k*, average pairwise sequence divergence; A, number of alleles; AR, allelic richness; *F*_IS_, inbreeding coefficient; *H*_*o*_, observed heterozygosity; *H*_*e*_, expected heterozygosity; *P* values are indicated for multilocus Hardy–Weinberg equilibrium tested against an alternative hypothesis of heterozygote deficit.

### Genetic structure

Founder signatures showing few unique haplotypes distinguish populations in the south, while northern populations differ more by haplotype frequency than by private haplotypes (Fig. 2, Table 3). The Lancashire population is comprised of a combination of shared and unique haplotypes, consistent with persistence of native and the survival of non-native lineages, introduced in 1913. Pairwise *F*_ST_ and Ф_ST_ values were significant for most comparisons among sampling locations, and highest for comparisons with Norfolk or Somerset (Tables 4 and 5). The results of the mtDNA SAMOVA analysis indicated significant population genetic structure for each assumed number of groups, from 2 to 10 (*P <* 0.00001 in each case; Table 6). Although the *F*_CT_ value was highest for seven groups, the major increase on *F*_CT_ occurred for three groups, with values only increasing slightly thereafter (Table 6).

**Table 4:** Pairwise *F*_ST_ (below diagonal) and Φ_ST_ (above diagonal) for roe deer between locations in the United Kingdom for 744 bp of the mtDNA control region

	1	2	3	4	5	6	7	8	9	10	11
1 Moray		0.01	**0.57**	**0.65**	**0.24**	**0.33**	**0.49**	**0.75**	**0.27**	**0.27**	**0.43**
2 Perth	0.03		**0.53**	**0.6**	**0.21**	**0.34**	**0.45**	**0.73**	**0.25**	**0.3**	**0.43**
3 Ayrshire	**0.41**	**0.33**		0.01	**0.2**	**0.4**	0.01	**0.86**	**0.25**	**0.73**	**0.79**
4 Carlisle	**0.45**	**0.37**	0.01		**0.34**	**0.57**	0.04	**0.97**	**0.37**	**0.82**	**0.94**
5 Durham	**0.11**	0.06	**0.19**	**0.25**		**0.24**	0.08	**0.87**	0	**0.55**	**0.7**
6 Lancashire	**0.17**	**0.15**	**0.28**	**0.36**	**0.14**		**0.36**	**0.85**	**0.23**	**0.55**	**0.66**
7 N York	**0.36**	**0.29**	0.01	−0.01	0.12	**0.28**		**0.89**	**0.15**	**0.71**	**0.81**
8 Norfolk	**0.64**	**0.63**	**0.81**	**0.92**	**0.84**	**0.78**	**0.89**		**0.83**	**0.86**	1
9 Berks	**0.24**	**0.21**	**0.19**	**0.26**	**0.16**	**0.15**	**0.2**	**0.79**		**0.47**	**0.6**
10 Dorset/Wiltshire	**0.33**	**0.35**	**0.56**	**0.61**	**0.45**	**0.37**	**0.57**	**0.73**	**0.35**		**0.15**
11 Somerset	**0.54**	**0.54**	**0.76**	**0.88**	**0.75**	**0.67**	**0.83**	**1**	**0.66**	**0.17**	

Values in bold indicate significance (*P* < 0.05).

**Table 5:** Pairwise values of *F*_ST_ using 16 microsatellite loci

	1	2	3	4	5	6	7	8	9	10	11	12
1 Moray												
2 Perth	**0.05**											
3 Ayr	**0.08**	**0.09**										
4 Carlisle	**0.07**	**0.08**	**0.03**									
5 Durham	**0.11**	**0.10**	**0.03**	**0.02**								
6 Lanc	**0.11**	**0.12**	**0.10**	**0.08**	**0.10**							
7 N York	**0.14**	**0.14**	**0.06**	**0.05**	**0.04**	**0.11**						
8 Norfolk	**0.18**	**0.19**	**0.20**	**0.20**	**0.22**	**0.19**	**0.24**					
9 Berks	**0.21**	**0.20**	**0.19**	**0.21**	**0.24**	**0.20**	**0.23**	**0.27**				
10 Dorset/Wiltshire	**0.22**	**0.20**	**0.21**	**0.22**	**0.25**	**0.24**	**0.23**	**0.27**	**0.13**			
11 Somer	**0.24**	**0.23**	**0.27**	**0.28**	**0.31**	**0.28**	**0.31**	**0.32**	**0.19**	**0.09**		
12 Glas	**0.03**	**0.02**	**0.05**	**0.06**	**0.09**	**0.08**	**0.11**	**0.19**	**0.14**	**0.18**	**0.24**	

Significant values following Bonferroni adjustment are in bold (*P* ≤ 0.003).

**Table 6:** Results from the spatial analysis of molecular variance (SAMOVA) showing values for variation among groups (*F*_CT_) and within populations (*F*_SC_)

*K*	Groupings	*F*_CT_	*F*_SC_
2	[Norfolk] [Moray, Perth, Ayr, Carlisle, Durham, N York, Berks, Lancs, Somerset, Dorset]	0.441	0.531
3	[Norfolk] [Moray, Perth, Somerset, Dorset] [Ayr, Carlisle, Dur, N York, Berks, Lancs]	0.565	0.267
4	[Norfolk] [Moray, Perth] [Ayr, Carlisle, Durham, N York, Berks, Lancs] [Somerset, Dorset]	0.592	0.176
5	[Norfolk] [Moray, Perth] [Ayr, Carlisle, Durham, N York, Berks] [Somerset, Dorset] [Lancs]	0.612	0.116
6	[Norfolk] [Moray, Perth] [Ayr, Carlisle, Durham, N York] [Berks] [Somerset, Dorset] [Lancs]	0.618	0.07
7	[Norfolk] [Moray, Perth] [Ayr, Carlisle, N York] [Durham] [Berks] [Somerset, Dorset] [Lancs]	0.623	0.031
8	[Norfolk] [Moray, Perth] [Ayr, Carlisle, N York] [Durham] [Berks] [Somerset] [Dorset] [Lancs]	0.613	0.036

**Figure 3:**
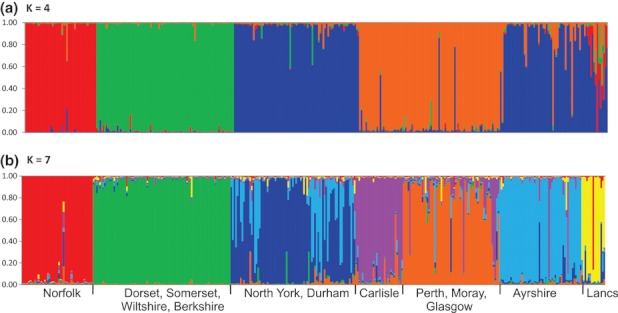
Assignment probabilities of individuals to putative population clusters at (a) *K* = 4 (b) *K* = 7 using the program STRUCTURE 2.3.2. Locations where individuals were sampled are indicated below the graph.

**Figure 4:**
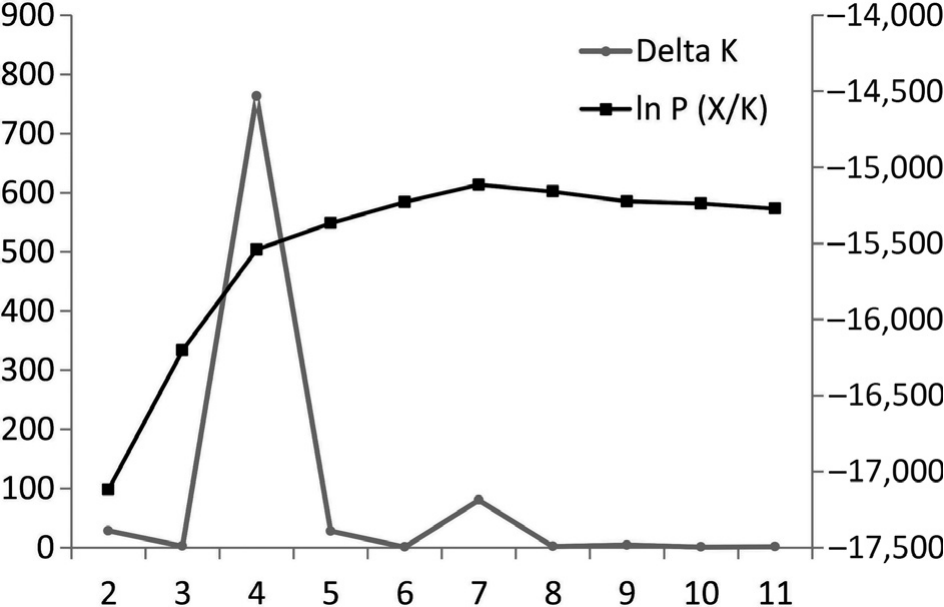
Posterior probability of the data (ln [*P*(*D*^|^*K*)]) and values of Δ*K* (Evanno et al. 2005) as a function of *K* (number of clusters), as resulting from the simulations in structure.

Analysis in STRUCTURE based on the 16 microsatellite DNA loci was broadly consistent with the results based on *F*_ST_ and the SAMOVA, although some proximate populations within a region that had shown significant *F*_ST_ comparisons were clustered by STRUCTURE (Fig. 3). The highest hierarchical level of structure, as indicated by Δ*K* (Evanno et al. 2005), was *K* = 4, with a second mode present at *K* = 7 (Fig. 4). The highest *K*, based on the Ln *P*(*X*^|^*K*) profile, was also at *K* = 7, supporting the distinction of Carlisle and Lancashire beyond those regions already supported when *K* = 4. A separate analysis including only samples from northern United Kingdom (Lancashire, N York, Durham, Carlisle, and Scotland) indicated that these furthers divisions are robust, but at a lower level of support compared with the rest (Fig. S1).

Using prior population information in STRUCTURE (*M* = 0.05), only three potential individuals with mixed ancestry were identified, one male and two females. The male was sampled from Perthshire and assigned to this population with 25% and to the North Yorkshire/Durham population with 36% probability. One of the females was sampled from the south west (Wiltshire) and assigned to this population with 39% probability, but also to the Lancashire population with 43% probability. The other female was sampled from Ayrshire and assigned to this population with 4% probability and to the Carlisle population with 81% probability.

**Figure 5:**
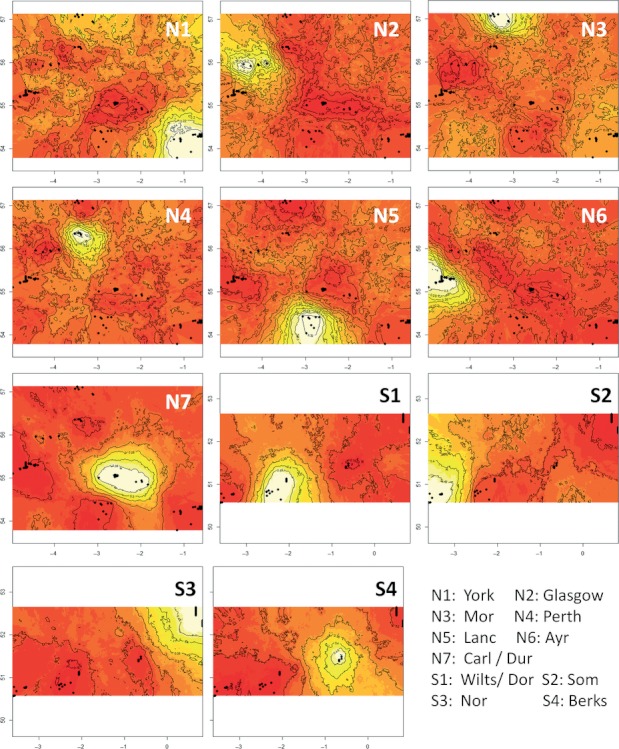
Results of geneland analyses showing posterior probabilities and spatial organizations of roe deer in northern (N1–N7) and southern (S1–S4) regions of mainland Britain.

**Figure 6:**
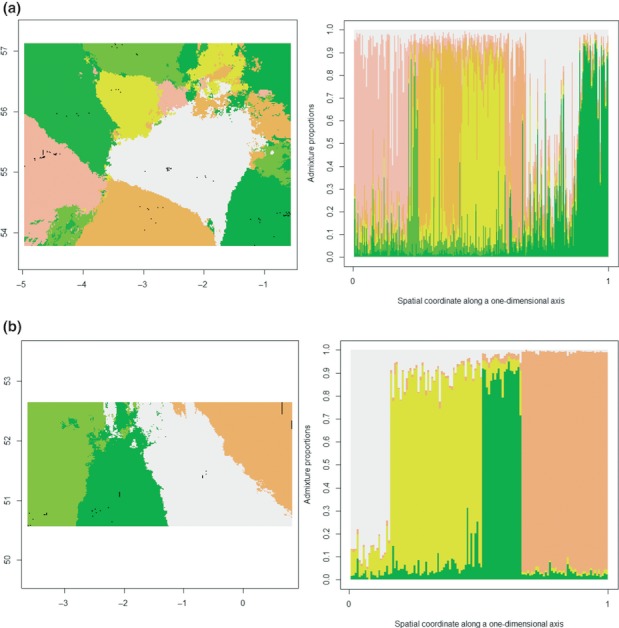
Posterior proportions of admixture inferred by Geneland for (a) northern and (b) southern populations.

**Figure 7:**
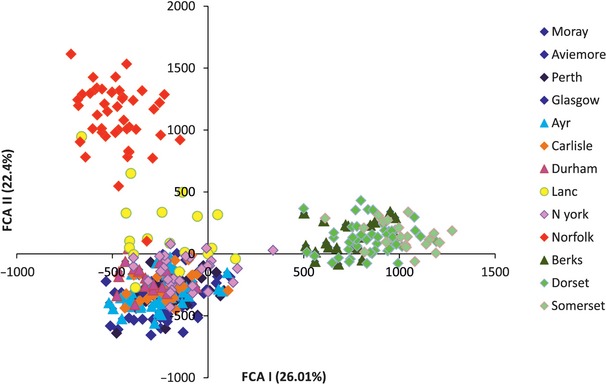
Factorial correspondence analysis (FCA) of population multilocus scores computed using GENETIX. Multilocus scores are computed in the bivariate space defined by the first two factorial components.

**Figure 8:**
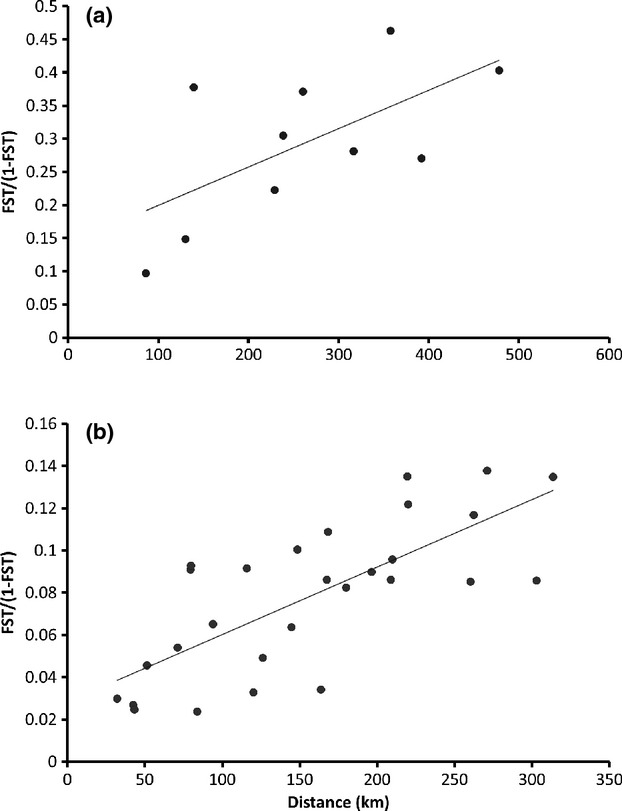
Isolation by distance tests for correlation between genetic differentiation (based on microsatellites) and geographic distance between (a) southern roe (*R*^2^ = 0.40, *P* > 0.05) and (b) northern roe based on microsatellites (*R*^2^ = 0.55, *P* < 0.001).

The results from Geneland showed the greatest degree of structure (a total of 11 putative populations, seven in the north and four in the south based on comparative likelihood outcomes among the eight runs; Fig. S2), with all sample sites being identified as separate clusters apart from the combination of Durham and Carlisle in the north and Dorset with Wiltshire in the south (Fig. 5). The assessment of admixture showed a higher degree among the northern than among the southern populations (Fig. 6). At the finest levels of subdivision, there was no complete agreement between methods. For example, STRUCTURE separated Carlisle when *K* = 7, but Geneland did not for *K* = 11. The FCA plot (Fig. 7) supported essentially the same four clusters as identified in STRUCTURE using Δ*K*. Mantel tests for correlation between genetic and geographical distance showed a nonsignificant positive trend for the southern populations (*P* > 0.05), and a significant pattern of isolation by distance for the northern populations (*P* < 0.001; Fig. 8), although the difference in the number of sites will have affected the significance of the regressions (and both show a positive trend).

## Discussion

### Genetic diversity

Levels of genetic variability diminished from north to south. Higher diversity in Scotland is consistent with the understanding, based on historical records, that a refuge population survived there while the southern populations were being extirpated. Low diversity in the south could reflect small founder populations introduced from Scotland and Europe. The intermediate level of diversity in some middle England populations may reflect a relatively recent (perhaps since medieval times when English populations were depleted) expansion of the remnant Scottish population, founding new populations as the range expanded and establishing a pattern of isolation by distance. This would also be consistent with the results from Structure analyses where those divisions are supported at a lower hierarchical level, and by the results in Geneland showing greater admixture across the northern range (Fig. 6).

In spite of the sometimes low mtDNA diversity in the recently founded populations in the south (e.g., no mtDNA diversity remaining in the Norfolk and Somerset populations) reflecting haplotype sampling in the small founder groups, overall levels of haplotype and nucleotide diversity (Table 3) were comparable with values reported for other roe deer populations in Europe (Lorenzini et al. 2002; Zachos et al. 2006). Microsatellite levels of variability were moderate (*H*_e_ = 0.59–0.62). In other local European roe deer populations, microsatellite diversity has ranged from low (*H*_e_ = 0.17–0.58; Lorenzini et al. 2002), to moderate (*H*_e_ = 0.63–0.66; Kuehn et al. 2004) and relatively high (*H*_e_ = 0.74–0.79; Zachos et al. 2006). An earlier study based on British roe deer reported very little polymorphism at allozyme loci (see Hewison 1995), but the difference may reflect the greater power available in markers based on sequence diversity and noncoding loci (see Zachos et al. 2006).

Consistent with this study, populations of white-tailed deer that experienced bottlenecks during introductions also retained appreciable levels of nuclear genetic diversity. This was attributed to the ability of their populations to expand very quickly (DeYoung et al. 2003) and thereby minimize loss by drift (see Gilpin and Soule, 1986). Demographic recovery is expected to be rapid for the roe deer, as they are known to be ecologically adaptable and able to exploit newly available habitat quickly (Putman and Langbein 2003). Many of the habitats into which roe deer were introduced encompassed newly forested sites, which could have enabled populations to thrive (Prior 1995). Furthermore, roe deer have a greater reproductive capacity than many other large mammals, as shown by the regular production of twins and an early age of first reproduction (Geist 1998). The most recent census data estimates that there are approximately 500,000 roe deer in Britain (Harris et al. 1995).

### Population structure

Wide ranging habitat generalists such as deer are often expected to exhibit low levels of population structure and a high potential for gene flow (Coltman 2008). Exceptions include species with a history of introductions and other types of human interference, such as white-tailed deer (DeYoung et al. 2003) and Alpine ibex (Biebach and Keller 2009). We chose the roe deer in Britain as a study system to further test the relative influence of anthropogenic and natural processes on the evolution of population structure in temperate zone ungulate species. This is facilitated in this system by the apparent historical removal of deer from much of their natural range in Britain (all areas in the south), together with the existence of a relatively undisturbed remnant population (in the north) and the reintroduction of roe deer into the south. If roe deer expanded out from their remnant or introduced founder populations as random mating populations, then we may expect to see three differentiated populations in Britain: one representing the remnant population in Scotland, one representing the founder population in southeast England, and the third representing a founder population in southwest England. This is somewhat complicated by further reintroductions (see Table 1); however, the genetic data suggest that most other translocation events into southwest England probably represented similar transfers from Scotland over a relatively short period of time (e.g., the relatively clear definition of population structure among four putative southern populations using Geneland).

If the three expanding populations had the opportunity to overlap, then admixture may be expected, which may be reflected in transition zones and patterns of isolation by distance across those boundaries. Historical data are incomplete; however, census data from 2000 and 2007 suggest that populations expanding (despite recent managed culls) out from reintroduction sites and from the remnant population in Scotland are only just now starting to overlap (Fig. 1). This is reflected in the high *F*_ST_ values for comparisons between Norfolk and Yorkshire or Berkshire (Fig. 1). There was a signal for isolation by distance, especially for the northern samples, which would be consistent with strong philopatry and a naturally expanding remnant population. In some cases, factors such as partial barriers to gene flow may be important. For example, high altitude areas were proposed to be inhibiting gene flow between populations of red deer (Haanes et al. 2010). Although there were instances where this may be relevant for roe deer (e.g., the North Yorkshire and Lancashire populations either side of the Pennines), there were also differentiated populations without apparent geographic barriers between them (Table 6, Figs. 3, 5).

In mainland Europe, various roe deer genetic studies have shown broad-scale population structure based on both mtDNA and microsatellite DNA data, defining three primary regions in western, central, and eastern Europe (e.g., Vernesi et al. 2002; Randi et al. 2004; Lorenzini and Lovari 2006). These regions reflect possible glacial refugial populations as described for various other species in Europe (see Hewitt 2000). The signature of local founder populations from translocations was evident in roe deer, especially in microsatellite DNA data (e.g., Randi et al. 2004; Thulin 2006). The broader trend was for structure at a relatively large geographic scale, although there was some apparently natural variation seen at a smaller scale across the Apennines (Randi et al. 2004), in central Italy (Vernesi et al. 2002), between northwestern and southern Spain (possibly related to refugial populations; Royo et al. 2007), and, to some extent, across Scandinavia (although much of this structure probably reflected local introductions; Thulin 2006).

In Britain, the larger scale pattern is clearly defined by the remnant population in Scotland, the European (German) introduction into Norfolk, and the translocation (or translocations) from Scotland to south western England (with this population probably differentiated by sampling effects at the time of the founder event or events). The smaller scale structure in Scotland and northern England is apparently largely due to natural processes, and suggests a strong tendency for philopatry and small natal dispersal range. Roe deer have quite small home ranges (often less than 100 ha), especially in fragmented habitat (see Cargnelutti et al. 2002; Coulon et al. [Bibr b10][Bibr b11]), and natal dispersal (at the age of 1–2 years) was found to rarely exceed a few km (Linnell et al. 1998; Coulon et al. [Bibr b10][Bibr b11]). After dispersal, roe deer show high levels of site fidelity, punctuated with relatively short-range (less than a few km) excursions (Danilkin and Hewison 1996; San Jose and Lovari 1998).

Consistent with this life history information, our data suggest limited dispersal in British roe deer. There was also little evidence that dispersal was male-biased. Discrepancies between the biparental (microsatellite) and uniparental (mtDNA) markers did not exceed the expected fourfold reduction in genetic structure (Prugnolle and de Meeus, 2002), which has been suggested to infer male sex-biased dispersal in other deer (Nussey et al. 2006). Furthermore, STRUCTURE did not show potential migrants to be biased toward either sex, although the number of putative migrants identified was very small. These results are consistent with recent genetic studies, which have reported roe deer to be highly sedentary, exhibiting little or no evidence for sex-biased dispersal (Coulon et al. 2006a; Gaillard et al. 2008; Bonnot et al. 2010). For example, in a study based on the fine-scale genetic structure of a roe deer population in France, analyses revealed that the spatial distributions of individuals were not random: adults of both sexes tended to be located spatially close to their relatives (Bonnot et al. 2010). Superimposed on a natural tendency for site fidelity in roe deer is an apparent further restriction to movement due to habitat fragmentation. For example, in highly fragmented habitats, genetic distance showed a closer correlation with urbanization than with geographic distance (Wang and Schreiber 2001). Therefore, an expected isolation by distance pattern generated by short-range dispersal may be disrupted by fragmentation, as seen in Europe due to agricultural practices or fragmented woodland (Coulon et al. 2004).

In Scotland and northern England, the strong pattern of isolation by distance over most of the range is disrupted somewhat by the higher *F*_ST_ values between Lancashire and neighboring sites (see Fig. 7). In 1913, a total of 12 roe deer were introduced into the population from Austria (see Table 1; reference to Windermere, Cumbria which is a distance <7 km from Lancashire sample sites), and the implication is that there was genetic integration, but this did not show a detectable influence on the nearby populations (even within 100 km). So integration was possible from the introduced deer (allowing for the possibility of genetic recovery), but the influence was felt only on a very small geographic scale, probably due to the insular behavior of the species.

In the region of south western England, there is also differentiation between sample sites, but these genetic distances are higher than seen in the north. It is possible that this pattern reflects a combination of both natural and anthropogenic processes involving multiple introductions together with differentiation by distance, although there are too few historical details about the introductions to allow a more careful assessment. If there had been unrecorded introductions from the continent, or even unknown surviving lineages from the native stock in the south, this may help explain the haplotypes unique to south western locations (e.g., Hap 1 & 2; see Fig. 2 and Table S2), although this could also simply reflect the sampling of rare haplotypes from the source population in Scotland. Barclay (1934) suggested that an ancient indigenous stock persisted in the southern English Petworth park, Sussex; although if true, it is unclear why introductions into this locality in the 1800s and 1890s would have been required (see Table 1). It is also unclear why any remnant population would not have retained greater similarity with the remnant population in Scotland. Taken together, the data suggest that surviving lineages in southern England are less likely to explain the observed patterns than the alternative of expanding founder populations, introduced from the north, although it remains a possibility. It is also possible that habitat fragmentation in the more urbanized south of England has contributed to the higher *F*_ST_ values seen between the sample sites there.

## Conclusion

Roe deer in Britain have evolved considerable population structure over a small remnant native range in Scotland and the north of England, all of which probably evolved in situ since the last ice age, given the geographic pattern of population sub-structuring. Introductions into habitat extirpated of roe deer in medieval times established populations that expanded quickly, but have not integrated with northern native populations for the most part. One likely exception is the introduction into an existing native population in Lancashire, but the influence of this admixture does not seem to have extended to neighboring populations, even though the geographic range is small. A possible exception may involve poorly documented introductions into south western England, but population structure is even more pronounced in this region. From the perspective of applied evolutionary inference, these data illustrate the importance of understanding the role of natural behavior associated with mating, dispersal, and habitat dependence when undertaking controlled management, and especially translocations. In this case, those behaviors meant that reintroductions established genetically depauperate (due to the founder effect) regional populations, or a very localized admixed population with limited impact on the larger population in Britain.

## Data Accessibility

DNA sequences: Genbank accessions JX971589–JX971615
Sample locations and microsatellite data: DRYAD entry doi:10.5061/dryad.v90p5

## Supporting Information

**Table S1.** Loci, primer sequences, and additional characteristics of 18 microsatellites selected to genotype roe deer. *Ref gives the source of the microsatellite.

**Table S2.** Distribution of mitochondrial haplotypes among the five roe deer populations studied, singletons marked in gray.

**Figure S1.** Assignment probabilities of individuals from northern locations only to putative population clusters at (a) *K* = 3, (b) *K* = 5 using the program STRUCTURE 2.3.2. Locations where individuals were sampled are indicated below graph (b).

**Figure S2.** MCMC iteration results in Geneland showing support for numbers of clusters for (a) full sample set, (b) northern subsample, and (c) southern subsample.
