# Supplementary material for: Evolution of population genetic structure of the British roe deer by natural and anthropogenic processes (Capreolus capreolus)
Source: Ecol Evol. 2013 Jan 10;3(1):89–102. doi: 10.1002/ece3.430 (PMC3568846; doi:10.1002/ece3.430)
Supplement: Supplementary file 1 [file ece30003-0089-SD3.doc]

**Supplementary Material**

Table S1. Loci, primer sequences and additional characteristics of 18 microsatellites selected to genotype roe deer. * Ref gives the source of the microsatellite.

| PCR no. | Ta | Locus | Primer Sequence | Label | Size range |
| --- | --- | --- | --- | --- | --- |
| PCR1 | 56.5 | MCM505^*1^ | ATC AGC ACC ATC TTA GGC CTA GA | HEX | 114-134 |
|  |  |  | TGT AGA TTC CCT CAA TAT AAA AAT GGT |  |  |
|  |  | MCM131^*1^ | ATT CAC AAA GCC GCG CTT G | HEX | 260-284 |
|  |  |  | ATC AAG CTC CCC TCT TCG GT |  |  |
|  |  | ILST011^*2^ | GCT TGC TAC ATG GAA AGT GC | FAM | 82-112 |
|  |  |  | CTA AAA TGC AGA GCC CTA CC |  |  |
| PCR2 | 54.7 | CSSM39^*3^ | AAT CGG AAC CTA GAA TAT TTT GAG | FAM | 178-186 |
|  |  |  | AGA TAA AAT GTG AGT GTG GTC TCC |  |  |
|  |  | IDVGA 8^*3^ | CTC TTG GGG GCG TGT TGT CT | HEX | 210-228 |
|  |  |  | TAG CAG AAA GCA CAG GAG TC |  |  |
|  |  | BM1706*3 | ACA GGA CGG TTT CTC CTT ATG | FAM | 238-250 |
|  |  |  | CTT GCA GTT TCC CAT ACA AGG |  |  |
|  |  | IDVGA29^*3^ | CCC ACA AGG TTA TCT ATC TCC AG | HEX | 142-148 |
|  |  |  | CCA AGA AGG TCC AAA GCA TCC AC |  |  |
| PCR3 | 62 | OarFCB304^*3^ | CCC TAG GAG CTT TCA ATA AAG AAT CGG | HEX | 150-182 |
|  |  |  | CGC TGC TGT CAA CTG GGT CAG GG |  |  |
|  |  | MAF70^*2^ | CAC GGA GTC ACA AAG AGT CAG ACC | NED | 130-152 |
|  |  |  | GCA GGA CTC TAC GGG GCC TTT GC |  |  |
|  |  | BM848^*3^ | TGG TTG GAA GGA AAA CTT GG | HEX | 356-368 |
|  |  |  | CCC TCT GCT CCT CAA GAC AC |  |  |
| PCR4 | 50 | NVHRT24^*4^ | CGT GAA TCT TAA CCA GGT CT | FAM | 132-140 |
|  |  |  | GGT CAG CTT CAT TTA GAA AC |  |  |
| PCR5 | 51.9 | HUJ117^*3^ | TCC ATC AAG TAT TTG AGT GCA A | HEX | 198-220 |
|  |  |  | ATA GCC CTA CCC ACT GTT TCT G |  |  |
|  |  | Bmc1009^*3^ | GCA CCA GCA GAG AGG ACA TT | NED | 280-292 |
|  |  |  | ACC GGC TAT TGT CCA TCT TG |  |  |
|  |  | RT1^*5^ | TGC CTT CTT TCA TCC AAC AA | NED | 222-240 |
|  |  |  | CAT CTT CCC ATC CTC TTT AC |  |  |
| PCR6 | 55.1 | CSSM43^*3^ | AAA ACT CTG GGA ACT TGA AAA CTA | HEX | 238-246 |
|  |  |  | GTT ACA AAT TTA AGA GAC AGA GTT |  |  |
|  |  | CSSM41^*3^ AAT TTC AAA GAA CCG TTA CAC AGC | | HEX | 120-124 |
|  |  |  | AAG GGA CTT GCA GGG ACT AAA ACA |  |  |
|  |  | Bm757^*3^ | TGG AAA CAA TGT AAA CCT GGG | NED | 172-204 |
|  |  |  | TTG AGC CAC CAA GGA ACC |  |  |
|  |  | NVHRT48^*3^ | CGT GAA TCT TAA CCA GGT CT | FAM | 86-90 |
|  |  |  | GGT CAG CTT CAT TTA GAA AC |  |  |
|  |  |  |  |  |  |

References: ^*1^ Hulme et al., ^*2^ Crawford et al., *^3^ Galan et al., ^*4^ Roed ^*5^ Wilson et al., .

Table S2. Distribution of mitochondrial haplotypes among the five roe deer populations studied, singletons marked in grey.

|  | **Dorset/ Wilts** | **Somerset** | **Berks** | **Norfolk** | **Moray** | **Perth** | **Ayr** | **N York** | **Durham** | **Carlisle** | **Lancs** | **∑** |
| --- | --- | --- | --- | --- | --- | --- | --- | --- | --- | --- | --- | --- |
| **Hap 1** | 7 |  |  |  |  |  |  |  |  |  |  | 7 |
| **Hap 2** | 28 | 19 | 2 |  |  |  |  |  |  |  |  | 49 |
| **Hap 3** |  |  |  | 41 |  |  |  |  |  |  |  | 41 |
| **Hap 4** | 8 |  | 7 |  | 2 |  |  |  |  |  |  | 17 |
| **Hap 5** |  |  | 10 |  | 2 | 7 | 40 | 20 | 7 | 25 | 4 | 115 |
| **Hap 6** |  |  |  |  |  | 4 |  |  |  |  |  | 4 |
| **Hap 7** |  |  |  |  | 12 | 13 |  | 3 | 5 | 1 |  | 34 |
| **Hap 8** |  |  |  |  |  | 7 |  |  |  |  |  | 7 |
| **Hap 9** |  |  |  |  |  |  |  |  | 1 | 1 |  | 2 |
| **Hap 10** |  |  |  |  |  |  |  |  |  |  | 4 | 4 |
| **Hap 11** |  |  |  |  |  |  |  |  |  |  | 2 | 2 |
| **Hap 12** |  |  |  |  | 2 | 1 |  |  |  |  |  | 3 |
| **Hap 13** |  |  |  |  | 3 | 2 | 4 |  |  |  |  | 9 |
| **Hap 14** |  |  |  |  | 3 |  |  |  |  |  |  | 3 |
| **Hap 15** |  |  |  |  | 2 |  |  |  |  |  |  | 2 |
| **Hap 16** |  |  |  |  | 2 |  |  |  |  |  |  | 2 |
| **Hap 17** |  |  |  |  |  |  | 2 |  |  |  |  | 2 |
| **Hap 18** |  |  |  |  |  |  |  |  |  |  | 1 | 1 |
| **Hap 19** |  |  |  |  |  |  |  |  |  |  | 1 | 1 |
| **Hap 20** |  |  |  |  |  |  |  |  |  | 1 |  | 1 |
| **Hap 21** |  |  |  |  |  | 1 |  |  |  |  |  | 1 |
| **Hap 22** |  |  |  |  | 1 |  |  |  |  |  |  | 1 |
| **Hap 23** |  |  |  |  |  |  |  | 1 |  |  |  | 1 |
| **Hap 24** |  |  |  |  |  |  | 1 |  |  |  |  | 1 |
| **Hap 25** |  |  |  |  |  |  | 1 |  |  |  |  | 1 |
| **Hap 26** |  |  |  |  |  |  | 1 |  |  |  |  | 1 |
| **Hap 27** |  |  |  |  |  |  | 1 |  |  |  |  | 1 |
| **∑** | 43 | 19 | 19 | 41 | 29 | 35 | 50 | 24 | 13 | 28 | 12 | **313** |

a)


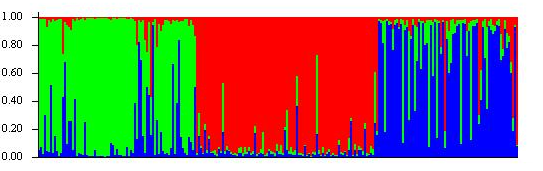


b)


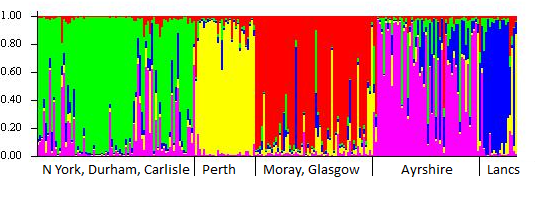


Figure S1. Assignment probabilities of individuals from northern locations only to putative population clusters at a) *K* = 3 b) *K* = 5 using the program STRUCTURE 2.3.2. Locations where individuals were sampled are indicated below graph b.

a)


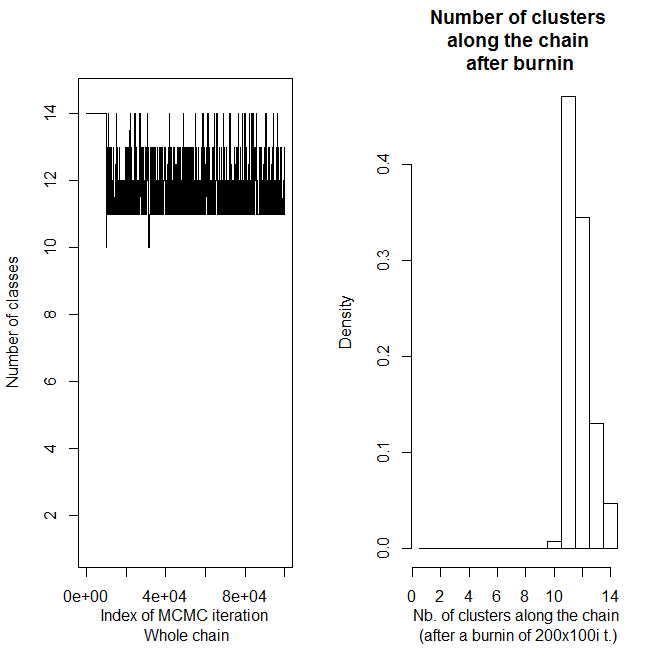


b)


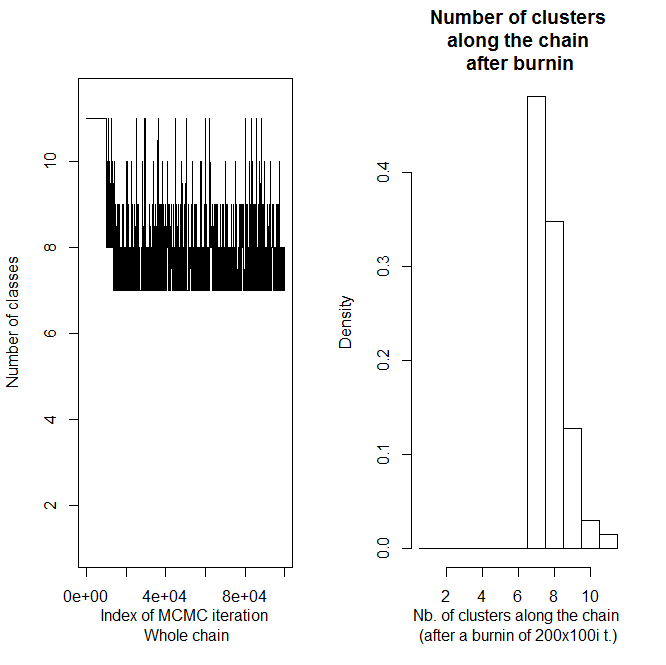


c)


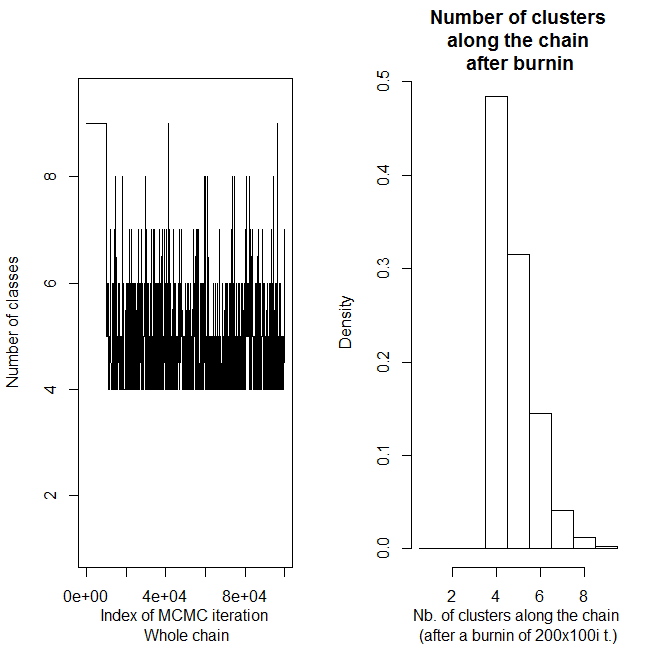


Figure S2: MCMC iteration results in Geneland showing support for numbers of clusters for a) full sample set, b) northern subsample, and c) southern subsample.
